# P05.34. Well-being and wellness behaviors among family medicine residents: an exploratory descriptive study

**DOI:** 10.1186/1472-6882-12-S1-P394

**Published:** 2012-06-12

**Authors:** A Brooks, S Dodds, M Guerrera, P Cook, R Benn, P Lebensohn

**Affiliations:** 1 University of Arizona, Tucson, USA; 2 University of Connecticut, School of Medicine, Hartford, USA; 3 University of Michigan Health System, Ann Arbor, USA

## Purpose

To present longitudinal changes on dimensions of well-being and wellness behaviors among residents in eight family medicine (FM) residencies.

## Methods

FM residents in the 2011 graduating class (n=56) were assessed at four time points, the beginning of each of the three years of residency and at graduation. Measures were self-administered online and included established measures of well-being: perceived stress, burnout (emotional exhaustion and depersonalization), emotional intelligence (empathy and perspective taking), depression, positive and negative affect, satisfaction with life, mindfulness, gratitude, and a measure of wellness behaviors (sleep, nutrition, physical activity, mind-body activities, being in nurturing relationships, being outdoors in nature, and alcohol use).

## Results

Perceived stress and positive affect were stable across PGY1, PGY2, and PGY3, but decreased significantly by graduation (p=.0005; p=.0011, respectively). Burnout (emotional exhaustion and depersonalization) increased significantly at the start of PGY2 and remained high through graduation (p=<0.0001). Emotional intelligence (empathy and perspective taking) decreased significantly from PGY2 to PGY3, but returned to PGY2 levels by graduation (p=<0.0001). Life satisfaction decreased in PGY2 and PGY3 but returned to PGY1 level by graduation (p=.0015). Depression, alcohol use, mindfulness, and gratitude remained stable throughout the residency. Two wellness behaviors showed significant increases across time, eating 5 servings of fruits and vegetables daily (p=.0005) and waking rested from sleep (p=.0433).

## Conclusion

Findings suggest that resident stress and distress persist throughout residency, but by graduation stress is reduced and positive affect, emotional intelligence, and life satisfaction return to improved levels from the baseline. Despite the rebound on these dimensions, the emotional exhaustion and depersonalization of burnout remained high at graduation. Only two wellness behaviors improved during the IMR residency, diet and sleep. These are preliminary results of a much larger study involving family medicine residents with and without a core curriculum in integrative medicine.

